# High rate of cardiac sarcoidosis presenting with cutaneous plaque type sarcoidosis in ^18^F-fluorodeoxyglucose positron emission tomography-computed tomography: a case series

**DOI:** 10.1186/1752-1947-8-17

**Published:** 2014-01-15

**Authors:** Satoshi Nakamura, Yoshio Hashimoto, Kaoru Nishi, Keiko Takeda, Toshihiro Mizumoto, Toshio Demitsu, Hajime Iizuka

**Affiliations:** 1 Department of Dermatology, Jichi Medical University, Saitama Medical Center, Tennumacho 1-847, Oomiya 330-8503, Saitama, Japan; 2 Department of Dermatology, Asahikawa Kousei Hospital, Asahikawa, 1-24, Hokkaido 078-8211, Hokkaido, Japan; 3 Department of Dermatology, Asahikawa Medical University, Midorigaokahigashi 2-1-1, 078-8510 Hokkaido, Japan

**Keywords:** Cutaneous sarcoidosis, Myocardial sarcoidosis, PET-CT

## Abstract

**Introduction:**

Myocardial sarcoidosis is known as a significant complication of sarcoidosis, but Holter electrocardiographic monitoring or echocardiograms might not be sensitive enough to detect cardiac involvement. While gallium scintigraphy has been recommended, ^18^F-fluorodeoxyglucose positron emission tomography-computed tomography might be more sensitive to detect sarcoidosis.

**Case presentations:**

This report comprises the cases of 12 Japanese patients. Two were male, and ten were female. Their age range was between 32 and 93 years. The average age of the patients was 63. We found internal involvement of sarcoidosis in eight (89%) of nine patients by positron emission tomography-computed tomography and in two (67%) of three patients by gallium scintigraphy. Myocardial sarcoidosis was detected in four (33%) of twelve patients, and specifically in three (75%) of four facial multiple plaque type sarcoidosis patients.

**Conclusion:**

The myocardial lesions detected by positron emission tomography-computed tomography could not be detected with conventional electrocardiogram or echogram. Positron emission tomography-computed tomography can detect sarcoid lesions of the whole body and is useful for the follow up of patients. We recommend positron emission tomography-computed tomography for those patients having cutaneous sarcoidosis, especially facial multiple plaque type sarcoidosis.

## Introduction

Sarcoidosis is a systemic granulomatous disease with involvement of various organs [1]. The various types of cutaneous sarcoidosis have been described previously, but the relation between cutaneous sarcoidosis and severity of general sarcoidosis has been shrouded in mystery [2].

Myocardial sarcoidosis is known as a significant complication, but Holter electrocardiographic monitoring and echocardiograms might not be sensitive enough to detect cardiac involvement [3]. Although gallium (^67^Ga) scintigraphy has been recommended as a diagnostic tool, ^18^F-fluorodeoxyglucose positron emission tomography-computed tomography (PET-CT) imaging might be more sensitive in detecting sarcoidosis [4]. Previous reports have suggested no significant difference between Ga scintigraphy and PET-CT [5]. Because myocardial sarcoidosis may result in unexpected sudden death [6], cardiac abnormalities should be adequately evaluated early in the course of the disease.

In this report, we investigated the relationship of cutaneous sarcoidosis and internal involvement, including myocardial sarcoidosis, using PET-CT and Ga scintigraphy. Among 12 patients, we found that 10 patients (83%) had internal involvement (Table 1). To the best of our knowledge, this report is the first to document that PET-CT is useful for detecting internal involvement in the cutaneous multiple plaque type of sarcoidosis.

**Table 1:** Patient demographics, affected areas, clinical types of sarcoidosis and complications

**Patient**	**Sex**	**Age (years)**	**Cutaneous sarcoid type**	**Imaging device used**	**Myocardial involvement**	**Other positive regions**	**Complications**
1	M	66	Plaque (face)	PET	+	Mediastinal LN diaphragm left parotid gland	Sjögren syndrome
2	F	74	Plaque (face)	PET	+	Mediastinal LN	Hepatitis C virus
3	F	93	Plaque (face)	PET	–	Left supraclavicular LN Mediastinal LN retroperitoneal LN	Lung tumor
4	F	48	Plaque (face)	PET	+	Mediastinal LN retroperitoneal LN Periaortal LN neck LN	Past history of lung tuberculosis Hepatitis B virus
5	F	63	Nodule (face)	Ga Scintigraphy	–	Muscle sarcoid (gastrocnemius)	Rheumatoid arthritis
							Sjögren syndrome
6	F	59	Nodule (face)	PET	–	Mediastinal LN	–
7	F	68	Nodule (face)	PET	–	Mediastinal LN right leg skin	Antinuclear antigen–positive (×1280)
8	F	58	Nodule (face)	Ga Scintigraphy	–	Mediastinal LN	Hashimoto's disease
9	F	32	Nodule (face, right knee)	PET	–	–	–
10	F	55	Scar (both knees)	PET	+	Mediastinal LN Left supraclavicular LN Retroperitoneal LN	–
11	F	43	Scar (both knees)	PET	–	Mediastinal LN	Myoma uteri
12	M	73	Subcutaneous type (face) nodule (left arm), papules (entire body)	Ga Scintigraphy	–	–	Past history of lung tuberculosis Hepatitis C virus

Ga, gallium; LN, lymph node; PET, positron emission tomography.

## Case presentations

### Patient 1

**Figure 1:**
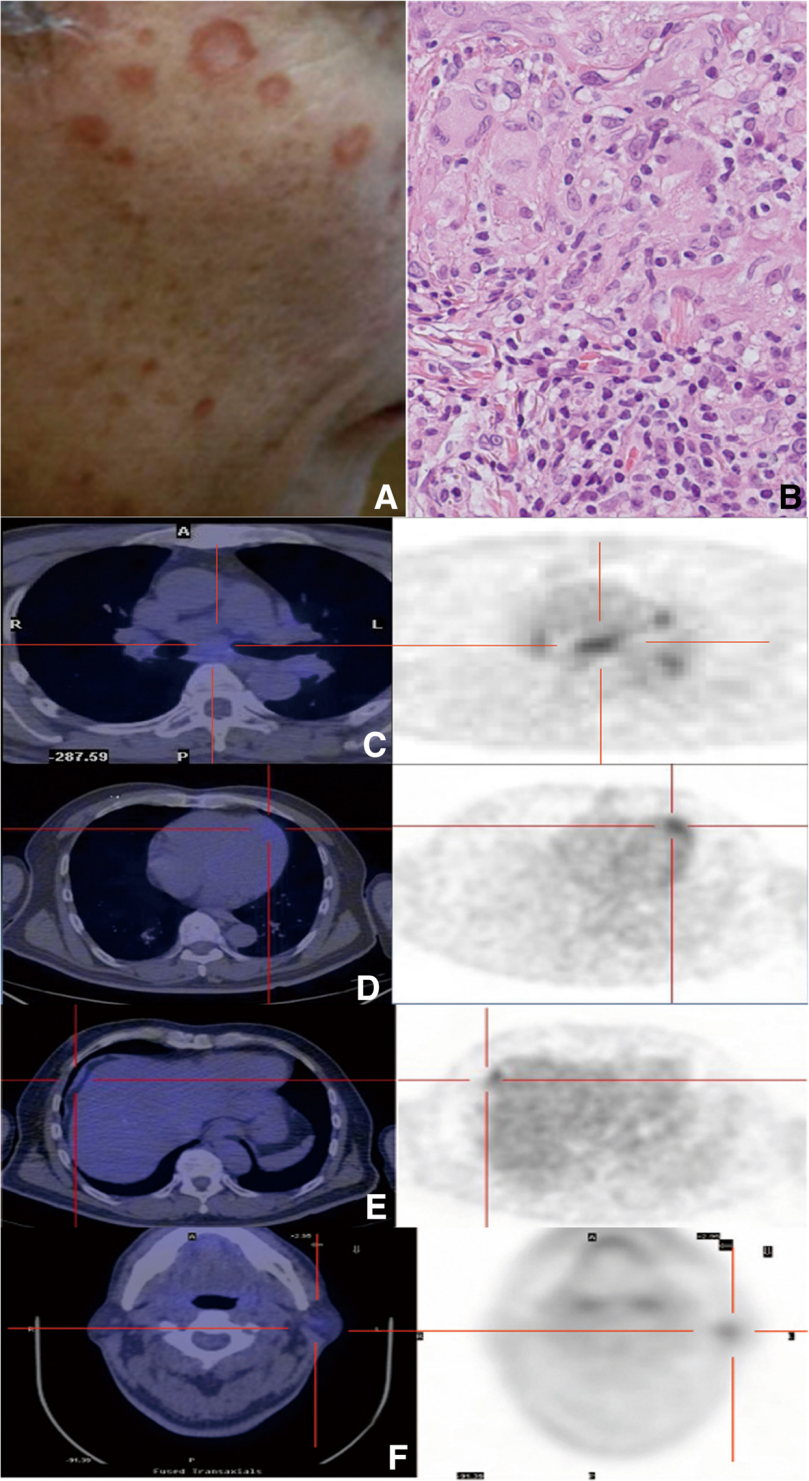
**Clinical appearance of patient 1. (A)** Up to 12 mm oval erythematous plaques on the patient’s face are shown. **(B)** Histopathological findings of patient 1 (hematoxylin and eosin stain, original magnification, ×100). Naked epithelioid cell granulomas accompanied by giant cells were observed in the upper dermis. **(C)** Positron emission tomography image of patient 1. Mediastinal and hilar lymph nodes show positive results. **(D)** Cardiac apex shows accumulation of ^18^F-fluorodeoxyglucose. **(E)** A positive result in the diaphragm is shown. **(F)** The left parotid gland was also positive.

A 66-year-old Japanese man with asymptomatic facial plaques of 6 months’ duration presented to our institution. He had no fever or arthralgia. Physical examination revealed up to 12mm oval erythematous plaques on his face (Figure 1A). His complete blood count, liver function tests and serum electrolytes were all normal. The anti-deoxyribonucleic acid (DNA) antibody and antinuclear antibody (ANA) were negative, but the anti-SSA antibody was positive. His serum immunoglobulin G was elevated to 2026mg/dl (normal, 870mg/dl to 1700mg/dl). His serum angiotensin-converting enzyme (ACE) level was normal (13.0IU/L; normal, 7.7IU/L to 29.4IU/L), but his serum lysozyme level was slightly elevated (11.5g/ml; normal, 4g/ml to 11g/ml). Histopathologically, naked epithelioid cell granulomas with giant cells were observed in the upper dermis (Figure 1B). Ophthalmologically, goniosynechia and retinal and choroid exudates were observed. Schirmer’s test was negative. Lip biopsy findings were consistent with Sjögren syndrome with lymphoid cell infiltration around the salivary gland. We diagnosed the patient with facial plaque type cutaneous sarcoidosis with Sjögren syndrome. PET-CT images revealed mediastinal and hilar LN swelling (Figure 1C), as well as involvement of the myocardium (Figure 1D), diaphragm (Figure 1E) and left parotid gland (Figure 1F). Cardiac echocardiographic and Holter electrocardiographic images did not show any abnormalities. A cardiovascular physician conducted follow-up of the patient for his cardiac involvement.

### Patient 2

**Figure 2:**
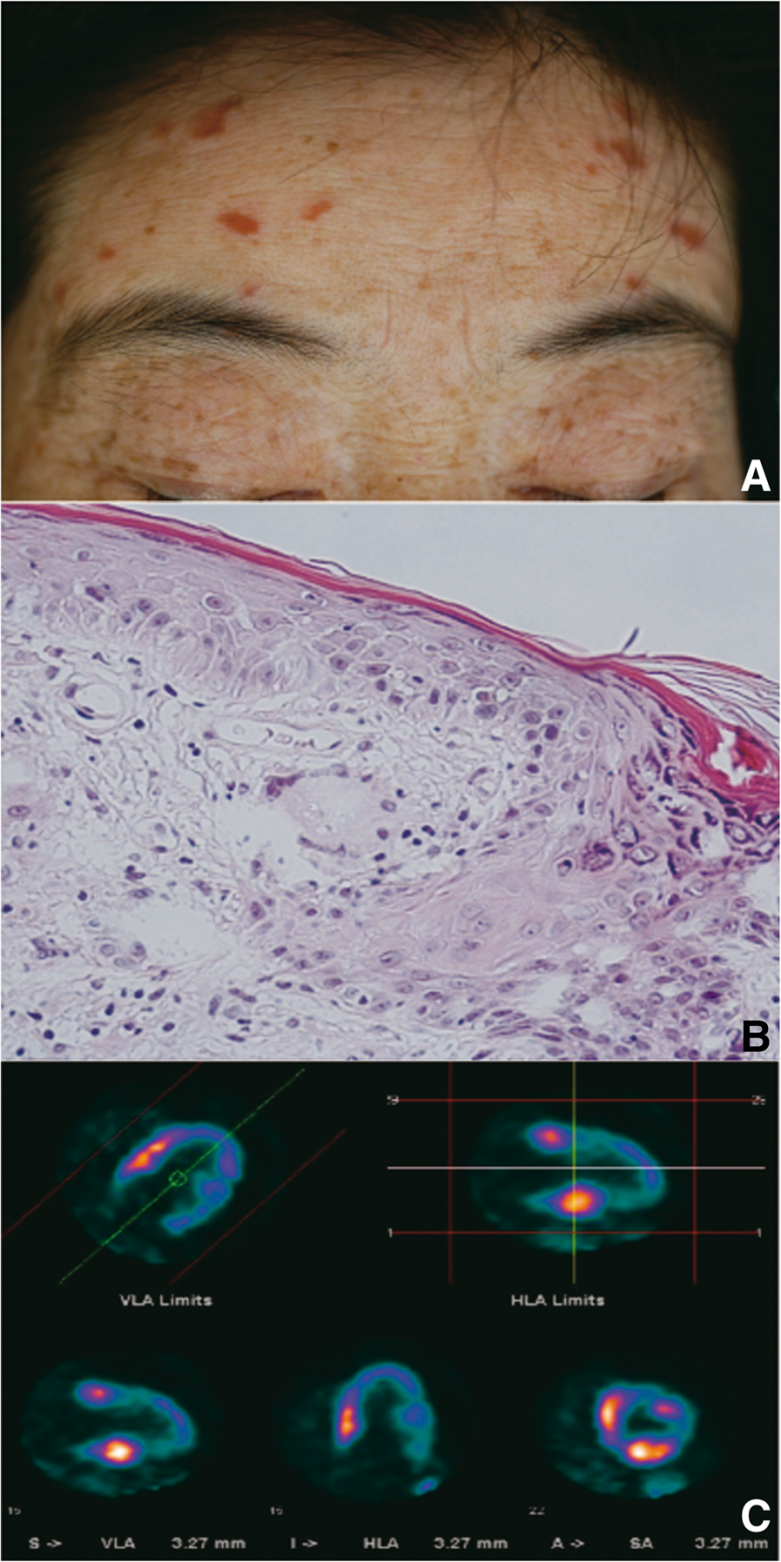
**Clinical appearance of patient 2. (A)** Five-millimeter, irregularly shaped plaques are shown. **(B)** Histopathological findings of patient 2 (hematoxylin and eosin stain; original magnification × 100). Naked epithelioid cell granuloma with giant cells can be seen in the upper dermis. **(C)** Positron emission tomography image of patient 2. Multiple myocardial focal uptakes are observed at the middle to inferior wall. HLA means horizontal long axis, VLA means vertical long axis, and SA means short axis in Figure 2-**C**.

A 74-year-old Japanese woman had asymptomatic plaques of 3 months’ duration. She had had hepatitis C virus for about 5 years. No palpitations, syncope history or arrhythmias were detected. Her physical examination revealed 5mm, irregularly shaped plaques on her face (Figure 2A). She had never received interferon therapy. Her complete blood count, liver function tests and serum electrolytes were all normal. ACE, lysozyme and ANA levels were also normal. Histopathologically, naked epithelial cell granuloma with giant cells were observed in the upper dermis (Figure 2B). PET-CT images revealed mediastinal and hilar lymph node (LN) swelling and multiple middle and inferior myocardial wall uptakes (Figure 2C). Her cardiac ejection fraction rate was 67%, and Holter electrocardiography revealed premature ventricular contractions and right bundle branch block. She was treated with 30mg prednisolone, and a pacemaker was implanted by a cardiovascular physician.

### Other patients’ symptoms

The other 12 patients were also Japanese. Two were male, and ten were female. Their ages ranged from 32 to 93 years. Their average age was 63 (Table 1). Among the 12 patients, 10 (83%) were found to have internal involvement. Internal involvement was found in eight (89%) of nine patients by PET-CT and in two (67%) of three by Ga scintigraphy. Cardiac involvement was found in four (33%) of the twelve patients. In the 12 patients, we found a predominance of myocardial sarcoidosis with multiple facial plaque type cutaneous sarcoidosis (75%) without subjective symptoms. Autoimmune-associated disease was found in four patients, and viral hepatitis was detected in three. In our series, two patients (17%) had a history of *Mycobacterium* infections with positive tuberculin tests and QuantiFERON® TB2G tests (Cellestis, Chadstone, Australia). We used special stains, including, Bacille Calmette-Guérin stain, Grocott’s methenamine silver stain, periodic acid–Schiff stain or Ziehl–Neelsen stain, to visualize acid-fast bacteria, but we could not find acid-fast bacteria, fungus or bacterial cutaneous infection in any of the biopsy samples. Cutaneous sarcoidosis was diagnosed on the basis of histopathology by the pathologist.

## Discussion

Sarcoidosis is a multisystem granulomatous disease of unknown etiology [1]. We performed screening for internal involvement by PET-CT and Ga scintigraphy in 12 cutaneous sarcoidosis patients. The results showed the usefulness of PET-CT for detection of internal involvement, especially for myocardial sarcoidosis. Our patients who had myocardial sarcoidosis did not show any subjective symptoms or any remarkable changes at the time the first electrocardiogram was taken. Because myocardial sarcoidosis may cause unexpected sudden death [6], cardiac abnormalities should be evaluated appropriately early in the course of the disease [4]. Although cardiac magnetic resonance imaging with gadolinium enhancement may be another approach, it detects only heart lesions [1-4]. PET-CT scans can allow clinicians to detect sarcoid lesions of the whole body and are useful for the follow-up of patients who receive pacemakers [1-4].

The various types of cutaneous sarcoidosis have been described previously, but the relation between cutaneous sarcoidosis and the severity of general sarcoidosis has been shrouded in mystery [2]. Our patient examinations revealed that myocardial sarcoidosis was more prevalent in patients with the multiple facial plaque type of cutaneous sarcoidosis (Table 1). On the one hand, the severity of myocardial sarcoidosis did not correlate with cutaneous sarcoidosis involvement (compare Figure 1A with Figure 2A). On the other hand, four patients had various multiple internal and LN involvements that were detected by PET-CT (Table 1), and three of these four patients also had myocardial sarcoidosis (75%). This pattern suggests that multiple lesions detected by PET-CT might be associated with myocardial sarcoidosis.

Sarcoidosis is accompanied by many complications, which include various autoimmune diseases that may cause lymphadenopathy [7,8]. Our patients had Sjögren syndrome, rheumatoid arthritis, Hashimoto disease and positive ANAs (×1280). This may be related to the immunopathogenic mechanism of sarcoidosis, which includes T-helper type 1 cell–related, uncontrolled, cell-mediated immunity.

A recent report showed the relation of sarcoidosis to hepatitis C viral infection [9]. One is triggered by antiviral therapies (75%), but the other is unrelated to the treatment (25%) [9]. The myocardial sarcoidosis was also associated with viral hepatitis in three cases (67%) without treatment by interferon. The significance of the relation of myocardial sarcoidosis and viral hepatitis remains to be determined. *Mycobacterium* and *Propionibacterium* have been identified as causative antigens of sarcoidosis [10]. The higher ratio of history of tuberculosis (17% vs. 9.2% of the same-age controls in 2009) might suggest the relationship between tuberculosis and sarcoidosis. In another report, Tchernev *et al.* suggested that the autoimmune etiology of sarcoidosis might occur through a process of molecular mimicry of infectious or other environmental antigens to host antigens [2].

## Conclusion

PET-CT can be used to detect sarcoid lesions of the whole body and is useful in patient follow-up. PET-CT has been shown to be quite useful for the detection of myocardial sarcoidosis, which was highly associated with the multiple facial plaque type of sarcoidosis. Although other associated involvements were noted in our patients, the significance in terms of the development of clinical sarcoidosis remains to be determined.

## Consent

Written informed consent was obtained from all of the patients for publication of this case report and any accompanying images. Copies of the written consents are available for review by the Editor-in-Chief of this journal.

## Abbreviations

ACE: Angiotensin-converting enzyme; ANA: Antinuclear antibody; LN: Lymph node; PET-CT: Positron emission tomography-computed tomography; HLA: horizontal long axis; VLA: vertical long axis; SA: short axis.

## Competing interests

The authors declare that they have no competing interests.

## Authors’ contributions

SN conceived of the study, participated in its design and coordination and drafted the manuscript. KN and KT participated in the design of the study and performed the statistical analysis. TM and YH conceived of the study. TD and HI drafted the manuscript and decided the results after discussion with SN. All authors read and approved the final manuscript.
